# A scoping methodological review of simulation studies comparing statistical and machine learning approaches to risk prediction for time-to-event data

**DOI:** 10.1186/s41512-022-00124-y

**Published:** 2022-06-02

**Authors:** Hayley Smith, Michael Sweeting, Tim Morris, Michael J. Crowther

**Affiliations:** 1 Department of Health Sciences, University of Leicester, Leicester, LE1 7RH UK; 2 Statistical Innovation, Oncology Biometrics, Oncology R&D, AstraZeneca, Cambridge, UK; 3 MRC Clinical Trials Unit at UCL, 90 High Holborn, London, WC1V 6LJ UK; 4 Department of Medical Epidemiology and Biostatistics, Karolinska Institutet, Stockholm, Sweden

**Keywords:** Machine learning, Prognostic modelling, Clinical risk prediction, Survival analysis, Simulation studies

## Abstract

**Background:**

There is substantial interest in the adaptation and application of so-called machine learning approaches to prognostic modelling of censored time-to-event data. These methods must be compared and evaluated against existing methods in a variety of scenarios to determine their predictive performance. A scoping review of how machine learning methods have been compared to traditional survival models is important to identify the comparisons that have been made and issues where they are lacking, biased towards one approach or misleading.

**Methods:**

We conducted a scoping review of research articles published between 1 January 2000 and 2 December 2020 using PubMed. Eligible articles were those that used simulation studies to compare statistical and machine learning methods for risk prediction with a time-to-event outcome in a medical/healthcare setting. We focus on data-generating mechanisms (DGMs), the methods that have been compared, the estimands of the simulation studies, and the performance measures used to evaluate them.

**Results:**

A total of ten articles were identified as eligible for the review. Six of the articles evaluated a method that was developed by the authors, four of which were machine learning methods, and the results almost always stated that this developed method’s performance was equivalent to or better than the other methods compared. Comparisons were often biased towards the novel approach, with the majority only comparing against a basic Cox proportional hazards model, and in scenarios where it is clear it would not perform well. In many of the articles reviewed, key information was unclear, such as the number of simulation repetitions and how performance measures were calculated.

**Conclusion:**

It is vital that method comparisons are unbiased and comprehensive, and this should be the goal even if realising it is difficult. Fully assessing how newly developed methods perform and how they compare to a variety of traditional statistical methods for prognostic modelling is imperative as these methods are already being applied in clinical contexts. Evaluations of the performance and usefulness of recently developed methods for risk prediction should be continued and reporting standards improved as these methods become increasingly popular.

**Supplementary Information:**

The online version contains supplementary material available at 10.1186/s41512-022-00124-y.

## Background

In a medical setting, we are often interested in the probability some health event occurs within a given time frame, for example, the probability of death within 5 years. We are sometimes interested in predicting, not only the probability that this event happens within a specified time frame, but also the rate of this event within given populations and how prognostic factors influence both the rate and probability of events. Prognostic models are designed to predict a clinical outcome, which can help make informed clinical decisions and treatment strategies and allow patients and families to put a clinical diagnosis into context [1]. Examples include estimating the probability that an individual will develop cardiovascular disease (CVD) over a given period to decide on statin prescription, or the probability of a patient with a new diagnosis of cancer will survive a given time.

The most commonly used method for the analysis of censored time-to-event data is the Cox proportional hazards model [2], which has been widely applied for prognostic modelling in healthcare [3, 4]. Even though it relies on the proportional hazards (PH) assumption, which assumes that the hazard rates for two individuals remain proportional over time, it is possible to relax this to allow for non-proportional hazards [5]. Methods have been developed to allow regression coefficients to depend on a function of time using, for example, restricted cubic splines [6] and fractional polynomial regression [7]. Incorporating interactions between covariates, variable selection techniques, and considering non-linear and/or time-dependent covariate effects is common practice when fitting a prognostic model [8]. These additions are all standard model building tools that can be incorporated into many statistical methods for risk prediction modelling. However, it may be easy to classify logistic regression as *statistics* and a random forest as *machine learning* but some would also describe lasso with a fixed penalty as statistics and lasso with a tuned penalty as machine learning. The latter methods are more similar to each other than to either of the former. Categorising methods as either machine learning or statistics is a complicated task yet these labels come with a certain research culture, in terms of terminology and approach to prediction tasks. Hence, we use these labels in order to focus the review and provide a distinction between the two approaches, which is discussed further in the “Methods” section of this review.

Machine learning methods are becoming increasingly popular within the medical field, in areas such as diagnostics, prognostics and drug discovery [9]. The typical description of these methods is that they exploit the amount of data available within electronic health records to identify complex relationships and patterns [10], due to their ability to model non-linear relationships and high-level interactions [11]. Machine learning methods have also been adapted to accommodate censored time-to-event data to offer flexible modelling of covariate effects [12] and handle high-dimensional survival data efficiently [10]. However, the potential benefits of machine learning over more traditional statistical methods for prognostic modelling are less clear in areas where the number of observations largely exceeds the number of variables [9]. A review conducted by Christodoulou et al. [13] found a lack of evidence to support the claim that machine learning methods perform better than logistic regression for clinical prediction modelling and that reporting standards of validation procedures were poor. Kantidakis et al. [14], however, concluded that both statistical and machine learning approaches can be useful when applied to survival data, though the advantages and disadvantages of any method should be presented. In addition, the interpretability of machine learning models varies depending on how complex the model is, with results from procedures such as neural networks being particularly difficult to interpret. Producing uninterpretable models could be considered a drawback of a particular method as it prevents understanding the underlying relationships within the data [15], which would be highly desirable, and arguably critical, in a medical setting. There has been increasing interest in researching explainable machine learning methods that can provide some interpretation of machine learning models [16], yet these model interpretations rely on human input to decipher what the explanation means often by assuming that the model ‘thinks’ in the same way we do [17]. When the goal of a model is to predict as accurately as possible and not parameter estimation, caution should be taken when explaining relationships between outcome and covariates. A standard mistake is to assign causal interpretations to parameters with no identification strategy, sometimes termed the ‘table 2 fallacy’ [18]. Because the conditioning required to identify an effect of one variable is different to another, aiming to make a single prognostic model ‘explainable’ is a fool’s errand.

Various new statistical and machine learning approaches are being developed and applied to health datasets to create prognostic models, with the separation between these two labels becoming more unclear [19]. It is vital that methods are evaluated and compared in multiple scenarios to highlight their advantages and disadvantages. Simulation studies are often used to compare existing and new methods in pre-specified scenarios [20]. An advantage of a simulation study is that the conditions under which the data are generated can be known, which allows us to evaluate a method’s performance in estimating the ‘truth’; this is not possible with a real-world clinical dataset, where performance is often assessed on a single held-back validation dataset. Simulation also enables methods to be compared and evaluated in multiple different settings, such as varying sample sizes or complexity of covariate relationships, to mimic aspects of real datasets of interest. By using simulation studies to assess the performance of prognostic modelling methods, further information can be gained about how these methods perform in different situations and how useful they may be in clinical settings. However, these comparisons must be fair and comprehensive; the DGMs need to be realistic [21], and the methods being compared should be applicable in real-world analyses.

This article aims to review simulation studies that compare statistical and machine learning methods for prognostic modelling. We qualitatively review which methods have been compared, the DGMs that have been used to evaluate them, and the methodology used to compare them in order to highlight issues and aspects that could be improved.

## Methods

### Statistical and machine learning methods

**Fig. 1:**
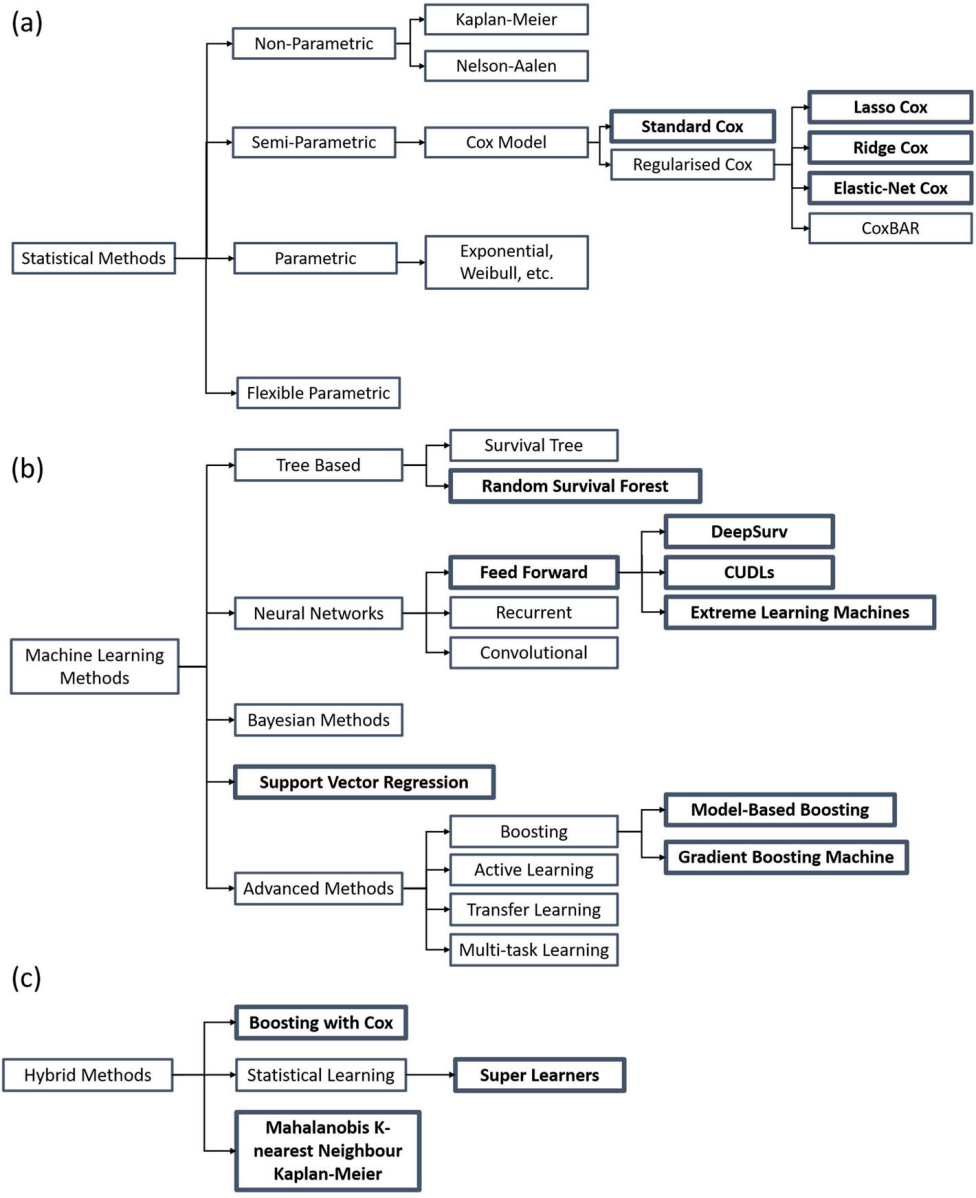
Taxonomy of methods for prognostic modelling as defined in this review, adapted from the taxonomy in Wang et al. [11]. Methods were categorised as statistical (**a**), machine learning (**b**), or hybrid methods (**c**) and highlighted in bold if included in articles in this review

We begin by defining how statistical and machine learning methods for risk prediction have been classified in this review. It is becoming increasingly difficult to delineate statistical and machine learning approaches. Breiman described two cultures of modelling: one which focuses on modelling the underlying data-generating processes and one which focuses on using algorithms to provide the most accurate predictions possible [19]. Similarly, Austin et al. [21] define ‘statistical learning’ as the use of parametric models for prediction and define ‘machine learning’ as the use of algorithms for prediction in their article. Parallel definitions are adopted for this review as many articles make a distinction between these two approaches; however, labelling methods as one approach or the other is not always helpful. In this review, statistical approaches are defined as those that focus on describing the underlying models through which the survival data are generated, for example, the Cox proportional hazards model. Machine learning approaches are defined as those that use algorithms to make predictions, without making any assumptions about the data, for example, neural networks. We also define in this review ‘hybrid methods’ referring to those that include elements of both machine learning and statistical approaches. For example, likelihood-based boosting and model-based boosting with the Cox model [22] combines the Cox model with boosting, a method commonly referred to as a machine learning approach. Wang et al. [11] described a taxonomy of statistical and machine learning methods, which we have adapted and present in Fig. 1 to detail how methods have been roughly categorised and labelled for the purpose of the review.

### Search strategy

A literature search was conducted to identify simulation studies that compare statistical methods to machine learning methods in a risk prediction setting for medical/healthcare data. Plasmode studies, a type of simulation study in which the covariate matrix is fixed and outcomes are simulated, were included in this literature search. Specific search criteria were used to search PubMed (date of search: 2 December 2020). PubMed was chosen as it largely hosts biomedical literature and so simulation studies comparing methods in non-medical settings should mostly be avoided. Articles were restricted to those that had been published between 1 January 2000 and 2 December 2020 with the full text available in English. The search string is available in the Appendix.

### Inclusion and exclusion criteria

**Fig. 2:**
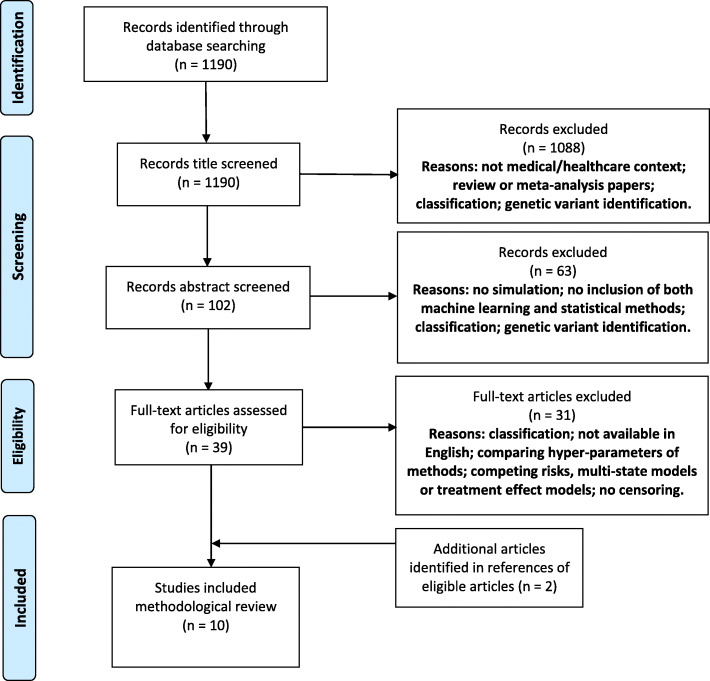
PRISMA flow diagram to illustrate the screening process

The inclusion criteria for this review are stated in Table 1. An article must have satisfied all of the inclusion criteria to be included in the review. The articles were first screened by title, then by abstract and finally by full-text. If it was unclear whether an article satisfied the inclusion criteria, it was automatically taken to the next stage of screening. A total of 1190 articles were identified from the search. The titles were screened resulting in 102 articles. These were then further screened by abstract to obtain a total of 39 articles. Full-text articles were obtained and reviewed in full, and an additional two articles were identified from the references of the eligible articles. These were not returned by the search as they were not available on PubMed. This resulted in a total of ten articles included in the review. The study identification journey and reasons for exclusion are shown in Fig. 2.

**Table 1:** Inclusion criteria used for the title, abstract and full-text screening

	Inclusion criteria *An article must satisfy all of the following criteria to be included in the review.*
1	Compare at least one machine learning method and at least one statistical method (according to our definitions). Any number of hybrid methods can be compared but a machine learning method and a statistical method must be included.
2	Methods included should be prognostic (risk prediction) models for one, specific outcome in a medical/healthcare context.
3	Methods included must be used to predict survival outcomes.
4	The simulation study must have been used to compare the methods with a time-to-event outcome with censoring.
5	Methods must be evaluated and compared in terms of prognostic ability.
6	Methods must not be for modelling treatment effects, feature selection or genetic variant identification.

### Data extraction

The intent of this scoping review was to collect methodological information qualitatively. Multiple aspects of the reviewed articles were of interest: the aims, DGMs, estimands/targets of analysis, methods used and performance measures. In this context, the estimands/targets are typically measures of prognostic performance. The main focus of this review was the DGMs used to simulate data and how complex this data was. Specifically, the distributions used to simulate survival times, the sample sizes, the number and type of covariates, relationships between covariates, such as correlations or interactions, and how censoring was simulated were all of interest. Information was also collected regarding the number of repetitions conducted and the justification for this number, what factors were varied between DGMs (e.g. comparing method performance across various sample size), how the simulated data was partitioned into training and testing sets, and how the methods were evaluated for performance. The results of the simulation studies were reviewed along with any other additional information, for example, if the article further evaluated the methods using a real dataset.

## Results

A total of ten articles were included in the review, published between 2000 and 2020. The authors and titles of these articles can be found in Table 2.

**Table 2:** Authors and titles of the articles included in this review

Author/s	Publication date	Title	Journal
Xiang et al. [23]	2000	Comparison of the performance of neural network methods and Cox regression for censored survival data	*Computational Statistics and Data Analysis*
Omurlu et al. [24]	2009	The comparisons of random survival forests and Cox regression analysis with simulation and an application related to breast cancer	*Expert Systems with Applications*
Lowsky et al. [25]	2012	A K-nearest neighbors survival probability prediction method	*Statistics in Medicine*
Geng et al. [26]	2014	A Model-Free Machine Learning Method for Risk Classification and Survival Probability Prediction	*Stat*
Gong et al. [27]	2018	Big Data Toolsets to Pharmacometrics: Application of Machine Learning for Time-to-Event Analysis	*Clinical and Translational Science*
Hu and Steingrimsson [28]	2018	Personalized Risk Prediction in Clinical Oncology Research: Applications and Practical Issues Using Survival Trees and Random Forests	*Journal of Biopharmaceutical Statistics*
Katzman et al. [29]	2018	DeepSurv: personalized treatment recommender system using a Cox proportional hazards deep neural network	*BMC Medical Research Methodology*
Wang and Li [30]	2019	Extreme learning machine Cox model for high-dimensional survival analysis	*Statistics in Medicine*
Golmakani and Polley [31]	2020	Super Learner for Survival Data Prediction	*International Journal of Biostatistics*
Steingrimsson and Morrison [32]	2020	Deep learning for survival outcomes	*Statistics in Medicine*

### Data-generating mechanisms and repetitions

#### Data-generating mechanisms

The number of DGMs used in each article ranged from 2 to 57 (median = 10.5). Two articles [25, 27] included DGMs where the simulated data was based on clinical data. All of the articles included at least one DGM where the PH assumption was true and five articles also included DGMs where the PH assumption was not true [23, 25, 26, 28, 32]. Four articles included high-dimensional data with large numbers of covariates [27, 30–32].

#### Repetitions

The number of repetitions per DGM in the simulation studies ranged from 1 to 1000 (median = 100 repetitions), whilst it was unclear in one study how many repetitions were used [30]. Two articles [29, 31] only simulated one dataset for each data-generating mechanism considered. Table 3 details the number of repetitions, the number of DGMs and what factors were changed for each of the DGMs (e.g. changing sample sizes or number of covariates included) for each article.

**Table 3:** The number of repetitions, number of data-generating mechanisms and factors varied in each article

	Repetitions	Factors varied in the data-generating mechanisms						
		Number of DGMs	Sample size	Failure time distribution	Number of covariates	Covariate relationships	Covariate effects	Censoring
Geng et al. (2014) [26]	100	20	✓	✓	✓	✓	✓	✓
Golmakani et al. (2020) [31]	1	6	✓		✓	✓	✓	
Gong et al. (2018)* [27]	500	57	✓		✓	✓	✓	✓
Hu and Steingrimsson (2018) [28]	1000	4	✓			✓	✓	
Katzman et al. (2018) [29]	1	2					✓	
Lowsky et al. (2012)** [25]	20	12	✓				✓	
Omurlu et al. (2009) [24]	1000	4	✓					
Steingrimsson and Morrison (2020) [32]	1000	16	✓	✓	✓		✓	✓
Wang and Li (2019) [30]	***	24		✓	✓			
Xiang et al. (2000) [23]	50	9	✓		✓	✓	✓	✓

*Gong et al. (2018) [27] also included three data-generating mechanisms where data was based on clinical data

**All simulated datasets in Lowsky et al. (2012) [25] were based on a real kidney transplant dataset

***Numbers of repetitions were unclear in Wang and Li (2019) [30]

#### Covariates

The number of covariates included in the simulated data ranged from two to 5000 (median = 40 covariates). Five articles [23, 27, 30–32] varied covariate numbers to evaluate the impact of additional and noise covariates. Covariates were simulated from Binomial, Bernoulli, Normal and Uniform distributions across the articles. Relationships between covariates included independent covariates (*N* = 5 studies) and correlated covariates (*N* = 4 studies). Five studies included DGMs that incorporated interactions between two or more covariates, i.e. x_1_*x_2_. Lowsky et al. [25] used 13 covariate values from a kidney transplant dataset. Table 4 provides a summary of the number and distribution of covariates and relationships between covariates included in each article.

**Table 4:** Number of covariates, distribution type and relationships between covariates in each article’s simulations

	Covariates								
	Number of covariates	Distribution				Relationships			
		Binomial	Normal	Uniform	Real Data	Independent	Correlation	Interaction, e.g. X_3 =_ X_1_X_2_	Correlation and interaction
Geng et al. (2014) [26]	2		✓				✓		✓
Golmakani et al. (2020) [31]	50, 1000		✓			✓		✓	
Gong et al. (2018)* [27]	2, 3, 250	✓	✓			✓		✓	✓
Hu and Steingrimsson (2018) [28]	50		✓				✓		✓
Katzman et al. (2018) [29]	10			✓		✓			
Lowsky et al. (2012)** [25]	13				✓				
Omurlu et al. (2009) [24]	5	✓		✓		✓			
Steingrimsson and Morrison (2020) [32]	30, 100		✓				✓		
Wang and Li (2019) [30]	500, 1000, 2000, 5000		✓				✓		
Xiang et al. (2000) [23]	2, 4	✓	✓			✓		✓	

*Gong et al. (2018) [27] used distributions and parameter values to model clinical data in their clinically relevant datasets and included three data-generating mechanisms where the covariate relationships were modelled to be clinically relevant

**Lowsky et al. (2012) [25] used real clinical data for their covariates and so exact relationships are unknown

#### Failure time simulation

Failure times were simulated from exponential, Weibull and Gamma distributions. The exponential model was the most common, with seven articles simulating failure times from this distribution for at least one DGM [23–25, 28, 29, 31, 32]. The DGM for simulating survival times in Geng et al. [26] was unclear. Covariate effects modelled on the log-hazard scale included null effects, linear effects, non-linear effects and time-dependent effects. Additionally, two articles [27, 30] transformed the covariates in some way, for example, applying a kernel. Table 5 provides a summary of the failure time distributions, assumptions and covariate effects for the DGM for each article.

**Table 5:** Failure time distributions, assumptions and covariate effects included in the data-generating mechanisms for each article

	Failure Times										
	Distribution			Assumptions			Covariate effects				
	Exponential	Weibull	Gamma	PH	PO	Non-PH	Null effects	Linear	Quadratic covariates	Non-linear	Time-dependent
Geng et al. (2014) [26]				✓	✓	✓ *		✓	✓		✓
Golmakani et al. (2020) [31]	✓			✓			✓	✓	✓		
Gong et al. (2018) [27]		✓		✓			✓	✓		✓ **	
Hu and Steingrimsson (2018) [28]	✓		✓	✓		✓	✓	✓			✓
Katzman et al. (2018) [29]	✓			✓				✓		✓ ***	
Lowsky et al. (2012)**** [25]	✓			✓		✓		✓			✓
Omurlu et al. (2009) [24]	✓			✓				✓			
Steingrimsson and Morrison (2020) [32]	✓		✓	✓		✓	✓	✓			✓
Wang and Li (2019) [30]		✓		✓				✓		✓ *****	
Xiang et al. (2000) [23]	✓			✓		✓		✓			✓

*Geng et al. (2014) [26] included a specific crossing hazards data-generating mechanism

**Gong et al. (2018) [27] take the exponential of the first covariate squared and cos transform second covariate; covariate coefficients were also obtained for the clinically relevant data-generating mechanisms by fitting each of the predefined models to clinical data

***Katzman et al. (2018) [29] use a Gaussian distribution for the linear predictor and include quadratic effects for both covariates

****Lowsky et al. (2012) [25] fit an exponential model to the clinical data to obtain estimates for the covariate coefficients to use in simulating the failure times

*****Wang and Li (2019) [30] transform the covariates by a radial basis kernel

#### Censoring

The level of censoring in each of the simulated datasets ranged from 0 to 75% (median = 27.5% censoring). Details of the censoring simulation were unclear in three articles [24, 25, 29]. A summary of how censoring was simulated in each of the articles can be found in Table 6.

**Table 6:** Level of censoring simulated and distribution of censoring times used in each article

	Censoring			
	Level of censoring (%)	Distribution		
		Uniform	Exponential	Other
Geng et al. (2014) [26]	15, 40	✓		
Golmakani et al. (2020) [31]	18		✓	
Gong et al. (2018) [27]	0, 25, 50, 75			✓*
Hu and Steingrimsson (2018) [28]	37		✓	
Katzman et al. (2018) [29]	Unclear			✓**
Lowsky et al. (2012) [25]	Unclear			✓***
Omurlu et al. (2009) [24]	Unclear			
Steingrimsson and Morrison (2020) [32]	18, 47	✓	✓	
Wang and Li (2019) [30]	25	✓		
Xiang et al. (2000) [23]	0, 20, 30, 50, 70		✓	

*Gong et al. (2018) [27] randomly chose if the time was a censoring time or event time

**Katzman et al. (2018) [29] included administrative censoring only

***Lowsky et al. (2012) [25] — censoring distribution was unclear

#### Training and testing datasets

Training dataset sample size ranged from just 50 to 7500 observations (median = 500 observations) and testing dataset sample size ranged from 50 to 13,525 observations (median = 600 observations) (Table 7). Training to test set ratios varied across the articles. Three articles, Geng et al. [26], Katzman et al. [29] and Lowsky et al. [25], also used validation datasets to select optimal hyperparameter values for the models. Nine articles obtained testing datasets (and validation datasets if used) by sampling from the same DGM as the training dataset; the models in these simulation studies were only internally validated. It was unclear how training and testing datasets were obtained in Omurlu et al. [24]. Sample sizes in some of the articles were very low. Small training sample sizes can highlight issues that may occur in clinical settings where rare outcomes are being studied or only small samples are available. However, for testing data, larger sample sizes or using cross-validation will produce more accurate estimations of performance measures within each repetition. Further discussion on the impact of data splitting techniques and training and testing sample sizes are beyond the scope of this review; however, this is discussed in the following papers [33–35].

**Table 7:** Training and testing data size and method used to split training and testing data

	Training and testing datasets		
	Training data size	Testing data size	Method
Geng et al. (2014)* [26]	100 200	1000 1000	Independent samples from DGM
Golmakani et al. (2020) [31]	450 720	50 80	10-fold cross-validation
Gong et al. (2018) [27]	200 400 500 600 800 1000	200 400 500 600 800 1000	Independent samples from DGM
Hu and Steingrimsson (2018) [28]	200 500	1000 1000	Independent samples from DGM
Katzman et al. (2018)* [29]	4000	1000	Independent samples from DGM
Lowsky et al. (2012)* [25]	500 1000 3000 7500	13525 13525 13525 13525	Independent samples from DGM
Omurlu et al. (2009) [24]	50 100 250 500	50 100 250 500	Unclear
Steingrimsson and Morrison (2020) [32]	250 500 1000 1500 3000	250 500 1000 1500 3000	Independent samples from DGM
Wang and Li (2019) [30]	150	150	Two-fold cross-validation
Xiang et al. (2000) [23]	100 200	100 200	Randomly split whole sample into equal training and testing sets

*These articles also included validation datasets

### Methods compared in the articles

Across the 10 articles reviewed, a total of 29 distinct methods were compared: four statistical methods, 22 machine learning methods and three hybrid methods. Six of the studies were evaluating a method that was developed by the authors: IPCW-wSVM [26], Super Learner algorithms [31], DeepSurv [29], Mahalanobis K-nearest neighbour Kaplan-Meier (MKNN-KM) [25], CUDLs [32] and ELMCoxBAR [30]. The results of these articles almost always stated that the developed method performed equivalently or better than the other methods compared. A summary of the methods included in each article can be found in Table 8, with further details in Supplementary Table 1.

**Table 8:** Statistical, hybrid and machine learning methods included in each of the articles

	Statistical methods				Hybrid methods			Machine learning methods			
	Cox PH	Penalised L1 Cox (Lasso)	Penalised L2 Cox (Ridge)	Elastic Net Cox	Cox Boost	Super Learners	Mahalanobis K-nearest neighbour Kaplan-Meier	RSF	Neural Network	Boosting	SVM
Geng et al. (2014) [26]	✓										✓ ^***+***^
Golmakani et al. (2020) [31]	✓	✓	✓	✓	✓	✓ ^***+***^		✓		✓	
Gong et al. (2018) [27]	✓							✓	✓		
Hu and Steingrimsson (2018) [28]	✓	✓						✓			
Katzman et al. (2018) [29]	✓							✓	✓ ^***+***^		
Lowsky et al. (2012) [25]	✓						✓ ^***+***^	✓			
Omurlu et al. (2009) [24]	✓							✓			
Steingrimsson and Morrison (2020) [32]	✓	✓						✓	✓ ^***+***^		
Wang and Li (2019) [30]		✓	✓		✓			✓	✓ ^***+***^		
Xiang et al. (2000) [23]	✓								✓		

^+^Methods that were developed by the authors of the papers

#### Statistical methods

In the ten articles reviewed, there were four statistical methods in total that were compared, all of which used the Cox model or penalised versions of the Cox model. Six articles [23–27, 29] only included a ‘standard’ Cox model, i.e. with no penalisation. Four articles [23, 28, 31, 32] included the true main effects, interactions and/or quadratic variables in the Cox model; three articles [26, 27, 30] did not include true main effects, interactions and/or quadratic variables; and three articles [24, 25, 29] were unclear regarding how the Cox model was fit to the data. None of the articles that evaluated non-proportional hazards [23, 25, 26, 28, 32] or non-linear covariate effects [27, 29, 30] included those time-dependent or non-linear effects in the Cox model.

#### Machine learning methods

The 22 machine learning methods could be categorised into four groups: Random Survival Forests (RSF), Neural Networks, Boosting and Support Vector Machines (SVM). The most common machine learning method was the Random Survival Forest, included in eight articles. Five articles compared some form of neural network [23, 27, 29, 30, 32]. The only example of support vector machines/regression was the inverse probability of censoring weighting procedure based on weighted support vector machines (IPCW-wSVM) developed and compared by Geng et al. [26]. Two boosting algorithms were compared in Golmakani et al. [31]: model-based boosting and gradient boosting machine [36].

#### Hybrid methods

There were three hybrid methods in total included across the articles: boosting with the Cox model [22], two Super Learner algorithms [31] and Mahalanobis K-nearest neighbour Kaplan-Meier [25]. The two Super Learner algorithms derived by Golmakani and Polley [31] were categorised as hybrid methods due to Super Learners being defined as a flexible approach to statistical learning [37]. Mahalanobis K-nearest neighbour Kaplan-Meier method [25] incorporates the k-nearest neighbour algorithm to make predictions for new, unseen observations using the Kaplan-Meier curve [38]. Boosting with the Cox model, included in two of the articles [30, 31], uses the boosting approach to estimate the Cox proportional hazards model.

### Estimands and performance measures

The estimands and performance measures for each of the articles can be found in Table 9.

**Table 9:** Estimands and performance measures for each of the article’s simulation studies

		Estimands				Performance measures		
	Selection for time *t*	S(t|x)	h(t|x)	Linear predictor: *η(****x****)*	Restricted Mean Survival Time (RMST)	MSPE	C-Index*	Integrated Brier Score
Geng et al. (2014) [26]	1/5th,…,5/6th quantiles of training survival times	✓				✓		
Golmakani et al. (2020) [31]	N/A			✓			✓	
Gong et al. (2018) [27]	Unclear	✓					✓	
Hu and Steingrimsson (2018) [28]	25th, 50th and 70th quantile of training marginal survival times	✓				✓		
Katzman et al. (2018) [29]	N/A for linear predictor; Unclear for restricted mean survival and C-index			✓	✓	✓	✓	
Lowsky et al. (2012) [25]	*T* = 5 with step size of 0.25	✓						✓ **
Omurlu et al. (2009)*** [24]							✓	
Steingrimsson and Morrison (2020) [32]	S(*t*)*: t* = median marginal failure time; RMST: *τ* = 85th quantile of marginal observed times	✓			✓	✓		
Wang and Li (2019) [30]	Unclear	✓	✓				✓	✓
Xiang et al. (2000) [23]	Unclear	✓					✓	

*A specified value for *t* for the C-index is not always required — if the model assumes proportional hazards then the C-index should remain the same regardless of time point

**Lowsky et al. (2012) [25] used the Integrated Brier Score with added inverse probability of censoring weights. This is referred to as the IPEC in the paper

***Omurlu et al. (2009) [24] were unclear in what the estimands were for their simulation study

#### Estimands

Seven articles estimated the survival probability [23, 25–28, 30, 32]. However, three of these [23, 27, 30] were unclear in what the specified value of time was when estimating the survival probability. Katzman et al. [29] and Golmakani and Polley [31] estimated the linear predictor in the proportional hazards model and Katzman et al. [29] further estimated the restricted mean survival time (RMST), though the value of time selected for this measure was not clear. The ELMCoxBAR method [30] estimated the hazard function for individual observations given their covariate values. The linear predictor and hazard function can be useful for discriminating between high- and low-risk individuals but less useful in terms of calibration and individual clinical decision-making unless transformed to a more tangible scale.

#### Prognostic performance measures

The prognostic performance measures can be separated into two categories: those that compare the model to the true underlying model and those that compare the model to the simulated data. No articles provided calibration plots, intercept or slope values.

##### Comparisons with the true model

Mean squared prognostic error (MSPE) is a measure of predictive accuracy, calculating the mean squared difference between the predicted values of an estimand and the true values calculated from the DGM. The MSPE integrates both bias and variance but their relative influence on the MSPE is dependent on sample size in the presence of bias [20]. Four articles evaluated MSPE across a range of survival times, aggregating or averaging the results [26, 28, 29, 32] and three of these articles evaluated this measure under various sample sizes [26, 28, 32].

##### Comparisons with the data

The Concordance Index (C-index) [39] is a measure of prognostic performance that compares the predictions made by the model to the observed data. It calculates the number of pairs of individuals in the testing dataset that are concordant over all possible pairs. A concordant pair is one in which the individual with the larger predicted survival probability also has the larger observed event/censoring time of the pair. It is commonly used as a measure of discrimination, for example, how well the model can distinguish between high-risk and low-risk individuals. Six articles used the C-index as a performance measure, calculating the average value over the simulation repetitions [23, 24, 27, 29, 31]; four of these included it as their only performance measure [23, 24, 27, 31]. Katzman et al. [29] provided confidence intervals for the average C-index obtained using bootstrapping and two articles [23, 30] included standard deviations for the average C-index. However, when a model does not assume proportional hazards, the value of the C-index will vary depending on the time point selected at which the estimand is evaluated [40]. All six articles that used the C-index [23, 24, 27, 29, 31] included methods in their comparisons that do not assume proportional hazards and so the C-index will vary dependent on the time point selected. These articles were also unclear in what time was chosen for these evaluations.

The Brier Score is the squared difference between the estimated survival probability of an individual and an indicator function of whether that individual is observed to have survived up to that time in the testing dataset, averaged over all individuals. The Integrated Brier Score (IBS) is then an overall measure of prediction at all times. Two articles, Wang and Li [30] and Lowsky et al. [25], used the IBS to measure model performance, providing box plots of the IBS over the simulation repetitions, with Lowsky et al. [25] accounting for censoring in the score by using the inverse probability of censoring weighting.

#### Simulation performance measures

Simulation performance measures quantify how model performance varies between simulated datasets. Three articles provided standard errors [26] or the standard deviation [23, 27] of their performance measures. Four articles [25, 28, 30, 32] included box plots of the IBS, the C-index and/or the MSPE to highlight the variation in these measures across datasets for each of the methods. None of the articles reported bias, coverage, or Monte Carlo standard errors for between-study simulation measures.

### Results of the articles

#### Proportional hazards assumption

The results of all nine of the articles that included the Cox model (with no penalisation) [23–29, 31, 32] showed that the Cox model outperformed or performed equally to all other methods when the proportional hazards assumption holds. In the five articles that evaluated DGMs where the hazards are non-proportional [23, 25, 26, 28, 32], the other methods compared outperformed the Cox model in at least one performance measure.

#### Varying sample sizes

Seven of the articles that varied training sample sizes [23–28, 31, 32] reported that sample size did not impact the relative performance of methods but did improve the performance of all methods. Lowsky et al. [25] reported that the MKNN-KM method only outperformed the Cox model when the sample size was small (*n* ≤ 1000). The impact of varying sample sizes on the performance of prognostic models is highly important in order to fully evaluate how these models perform. In a simulation study by Wallisch et al. [41], it was concluded that so long as the sample size is large, statistical and machine learning models have similar predictive accuracy in predicting cardiovascular outcomes.

#### High-dimensional settings

Golmakani and Polley [31] reported that the Cox Ridge, Gradient Boosting Machine and RSF methods did not perform as well as the Cox model, CoxBoost and Superlearner algorithms in high-dimensional settings. Conversely, Gong et al. [27] reported that the Cox model ‘failed to provide reasonable estimates’ due to the datasets having more covariates than observations but RSF and neural network methods had similar performance to low-dimensional settings. Steingrimsson and Morrison [32] found that the Doubly-Robust and Buckley-James RSFs had improved relative performance in settings with larger number of covariates. Wang et al. [30] reported that the trends of performance measures were similar in both high- and low-dimensional settings.

### Conclusions of the articles

Geng et al. [26] concluded that though the Cox model performs well when the true model does not deviate from the PH assumption, their IPCW-wSVM model is more flexible. Similarly, both Steingrimsson and Morrison [32] and Lowsky et al. [25] concluded that CUDLs and MKNN-KM, respectively, only outperform the Cox model when the PH assumption is not true. Hu and Steingrimsson [28] also concluded that RSFs could be preferable to the Cox model as this method avoids decreased performance due to model misspecifications. However, Gong et al. [27] concluded that though machine learning methods can be flexible and reliable, this depends on model selection and hyperparameter tuning and, equally, Xiang et al. [23] concluded that neural networks can be effective for survival but are highly variable depending on the dataset.

Golmakani and Polley [31] concluded that their Super Learner algorithms always perform as well or better in all the scenarios evaluated, including low- and high-dimensional datasets and Katzman et al. [29] concluded that DeepSurv is superior to the Cox model at modelling true covariate functions. Wang and Li [30] concluded ELMCoxBAR performs well when the correct kernel is used but is comparable to Penalized Cox models when the incorrect kernel is specified. Omurlu et al. [24] concluded RSF splitting rules all perform similarly and all methods had similar C-indexes.

### Additional information

Five articles [25, 26, 28, 29, 31, 32] went on to evaluate the methods using clinical datasets, including oncology datasets, genetic/gene expression datasets and transplant datasets. Katzman et al. [29] further evaluated DeepSurv as a treatment recommender using both simulated and real data as well as additional evaluations of predictive performance compared to Random Survival Forests and the Cox model using three clinical datasets.

## Discussion

This article has reviewed simulation studies that compare statistical and machine learning methods for risk prediction. In particular, which methods were compared and the methodology used to compare them was of interest in order to provide an overview of what research has been done to date and areas that may require improvement.

The key findings from this review were the limited number of articles identified that have compared statistical and machine learning methods using simulation studies, the lack of statistical methods compared in these articles and the poor reporting standards found. Only ten articles were identified; we view this as limited due to the abundance of articles that compare and evaluate methods which fall under one category only, for example, comparing the prognostic performance of statistical methods. There was a lack of statistical methods in the articles as they tended to focus more on the machine learning methods and statistical methods only included the Cox model. Interaction terms and non-linear covariate effects present in the DGM were often not included in the Cox model, nor did any study relax the PH assumption by modelling time-dependent effects. Incorporating interactions, variable selection, non-linear and/or time-dependent covariate effects are common extensions to the standard Cox model [8] and, by excluding these, the Cox model is expected to perform poorly in more complex data scenarios. In addition, many model building strategies exist to systematically select predictors and their effects for survival data [42] such as fractional polynomials [43], restricted cubic splines [44] and flexible backwards elimination approaches [5]. These are all standard modelling approaches conducted by the applied statistician which were not included in these articles (though at the time of publication some of these approaches were admittedly less well understood).

The reporting standard of many of the articles was poor. Key information, such as DGMs, estimands and how methods were implemented, was often missing or unclear making the studies difficult to reproduce. The selection of time points to evaluate estimands and/or performance measures was frequently not reported, for example, the time at which the survival probability was estimated. The C-index, for example, will vary depending on the time selected unless the PH assumption holds, which was not the case for the majority of methods included in these articles. In these situations, the time-dependent C-index [40] or the time-dependent AUC [45–47] should be used to ensure the measure captures the discriminative performance of the methods over time. The reporting standards of studies both comparing methods and/or reporting prognostic models have been questioned by multiple reviews, concluding that standards need to be raised [13, 20, 48–52]. Guidance regarding the best practices of reporting both simulation studies [20, 49] and reporting developed models for prognostic prediction for both statistical methods [53] and machine learning methods [54] are available and/or currently being developed. These guidance papers should be consulted to ensure high-quality designing, reporting and reproducibility of comparison studies.

Four of the five articles that used the C-index included it as their only performance measure. As the C-index is concerned with the ranking of predictions, the order could be correct but the predictions themselves could be miscalibrated. Accurate predictions are essential when applying these methods to medical contexts especially where decisions are taken based on absolute values of a prediction (e.g. prescription of statins according to predicted 10-year CVD risk ≥7.5%, as recommended by 2013 ACC/AHA guidelines) [55]. Including performance measures that evaluate both the discrimination and calibration of the methods would provide more detailed information on overall performance and clinical implications in medical settings.

This review highlights the difficulty of designing simulation studies that allow a fair comparison of methods. A data-generating mechanism that may be favourable for one method, such as a high-dimensional (*p* > *n*) setting where many machine learning methods are designed to excel, may be less appropriate for other methods unless statistical variable selection techniques are incorporated [56]. This was an issue in many of the articles reviewed; it was often reported that the Cox model failed to converge due to the high number of covariates and limited sample size. In these situations, traditional statistical methods are expected to fail unless variable selection or penalisation methods are considered which are, again, approaches that would be commonly used by applied statisticians and data scientists working with high-dimensional data. Similarly, a DGM with few covariates and uncomplicated data structure (e.g. no interactions or non-linearities) may favour a simpler statistical model. To avoid the issue of DGMs favouring one approach in simulation studies, Austin et al. [21] compared six estimation methods in a simulation study with six DGMs; outcomes from each DGM being simulated from one of the methods. In this case, every method would be expected to be ‘optimal’ for at least one of the DGMs in the simulation study, and both internal and external validity can be assessed across a range of methods. Researchers need to be mindful of not only data-generating mechanisms that may make a simulation study biased towards one approach, but also how their knowledge of particular methods may affect their implementation. Gelman [57] described the ‘methodological attribution problem’ which is highly relevant when comparing multiple methods; it is important to recognise how the researcher’s field of expertise will influence the implementation of methods [58]. For example, an applied statistician proficient in fitting the Cox model will have the knowledge to incorporate complex covariate effects but may not be as confident in tuning the hyperparameters of a neural network. Methods may not be implemented in a way that highlights true performance, which is certainly the case with the Cox model, and all of the methods compared where default hyperparameters were chosen, in the articles reviewed. Collaboration between researchers is key to ensuring methods are implemented to their fullest potential and comparisons are neutral when assessing performance [58].

It is important to note that statistical and machine learning approaches are very difficult to separate into two distinct categories. A distinction was made in this article in order to focus the scope of the review and as many articles do classify methods into these two approaches, though the usefulness of continuing to separate methods is debatable as these two cultures merge closer together. It is really the elements that make up the approaches we are interested in, for example, it may be the incorporation of regularisation or non-PH assumptions that improve prognostic performance and these are elements that could help with a ‘statistical’ method as much as a ‘machine learning’ method. Labelling methods as one approach or the other can lead to preconceptions about the performance and interpretability of the resulting models; discontinuing to classify methods may lead to more fair comparisons.

There are several limitations to this review. Firstly, PubMed was the only database used for the search and so articles could have been missed that would have fit the eligibility criteria. PubMed was chosen as this review focused on risk prediction in a medical context; however, a larger review on multiple databases, for example Web of Science, could be useful to review all simulation studies conducted to compare machine learning methods to statistical methods for prognostic modelling. Secondly, more complex survival analysis scenarios, such as competing risks and multi-state models, were excluded and we focused on simulation studies only. Many more articles could have been returned that have evaluated and compared methods using clinical data. Further reviews on these articles would be useful to analyse how machine learning methods and statistical methods are being compared overall, not just in a simulation setting.

The performance of machine learning methods compared to traditional statistical methods for risk prediction is still unclear. Kantidakis et al. [14] concluded that though Random Survival Forests and neural networks performed better than the Cox model in terms of the C-index and Integrated Brier Score, respectively, both of the machine learning methods required a longer computation time and the Cox model was easier to implement and interpret. Similarly, Allan et al. [59] reviewed methods for CVD prediction and concluded that although machine learning methods were reported to perform better than statistical methods, the quality of reporting was questionable, and Christodoulou et al. [13] found no benefit of machine learning methods over logistic regression for classification problems. It is important that better research is conducted to compare newly developed methods to the existing and commonly used statistical methods for risk prediction and that these comparisons are fair, comprehensive and well-reported.

## Conclusion

Assessing the accuracy of predictions for survival data using newly developed and existing methods is important in both simple and complex scenarios. Comprehensive and fair comparisons of methods’ performance, and usefulness in clinical settings, should be conducted using both simulation studies under a wide variety of DGMs and with clinical data. Including statistical methods that are commonly used by the applied statistician, reducing bias in method evaluations and ensuring high reporting standards would allow for more confident conclusions regarding model performance. We recommend that future comparison simulation studies (1) are conducted independently of developing a new method; (2) assess both discrimination and calibration, and not discrimination alone; (3) report variations in performance measures along with mean values; and (4) consider the ‘fairness’ of the comparison particularly with respect to the authors’ expertise in implementing different methods.

### Supplementary Information

**Additional file 1: Supplementary Table 1.** Statistical, machine learning and hybrid methods compared in each of the articles.

## Data Availability

Data sharing is not applicable to this article as no datasets were generated or analysed during the current study.

## Appendix

Search string used for PubMed search:

(((“machine learning”[Title/Abstract] OR “ai”[Title/Abstract] OR “ml”[Title/Abstract] OR “artificial intelligence”[Title/Abstract] OR “neural network*”[Title/Abstract] OR “ann*”[Title/Abstract] OR “deep learning”[Title /Abstract] OR “random forest*”[Title/Abstract] OR “random survival forest*”[Title/Abstract] OR “bayesian learning”[Title/Abstract] OR “bayesian network*”[Title/Abstract] OR “support vector*”[Title/Abstract] OR “svm”[Title/Abstract] OR “svms”[Title/Abstract])

AND (“survival”[Title/Abstract] OR “hazard”[Title/Abstract] OR “risk”[Title/Abstract] OR “prognos*”[Title/Abstract] OR “time to event”[Title/Abstract] OR “censor*”[Title/Abstract] OR “cox”[Title/Abstract] OR “kaplan*”[Title/Abstract] OR “spline*”[Title/Abstract] ))

AND (“simulate”[Title/Abstract] OR “simulation”[Title/Abstract] OR “simulations”[Title/Abstract] OR “simulated”[Title/Abstract]))

## Abbreviations

DGM: Data-generating mechanism
CVD: Cardiovascular disease
PH: Proportional hazards
RSF: Random survival forest
SVM: Support vector machine
MKNN-KM: Mahalanobis K-nearest neighbour Kaplan-Meier
IPCW-wSVM: Inverse probability of censoring weighting weighted support vector machine
RSMT: Restricted mean survival time
MSPE: Mean squared prediction error
C-index: Concordance Index
IBS: Integrated Brier Score
