# Supplementary material for: A scoping methodological review of simulation studies comparing statistical and machine learning approaches to risk prediction for time-to-event data
Source: Diagn Progn Res. 2022 Jun 2;6:10. doi: 10.1186/s41512-022-00124-y (PMC9161606; doi:10.1186/s41512-022-00124-y)
Supplement: Supplementary file 1 — Additional file 1: Supplementary Table 1. Statistical, machine learning and hybrid methods compared in each of the articles. [file 41512_2022_124_MOESM1_ESM.docx]

**Supplementary Material**

Supplementary Table 1: Statistical, machine learning and hybrid methods compared in each of the articles.

|  | Methods | | |
| --- | --- | --- | --- |
|  | Statistical Methods | Machine Learning Methods | Hybrid Methods |
| Geng et al. (2014) | - Cox Model | - IPCW-wSVM ***⁺*** |  |
| Golmakani et al. (2020) | - Cox Model - Penalized L1 Cox - Penalized L2 Cox - Elastic-Net Cox - Cox – true covariates | - Model-Based Boosting - Gradient Boosting machine - RSF log-rank | - SuperLearner algorithms ******⁺*** - CoxBoost |
| Gong et al. (2018) | - Cox Model | - RSF log-rank - Partial logistic ANN |  |
| Hu and Steingrimsson (2018) | - Cox Model – true main effects and interactions - Cox Model – true main effects only - Penalized L1 Cox Model | - RSF log-rank - RSF with feature selection - Conditional Inference Forest |  |
| Katzman et al. (2018) | - Cox Model | - DeepSurv***⁺*** - RSF log-rank |  |
| Lowsky et al. (2012) | - Cox Model | - RSF log-rank | - Mahalanobis K-nearest neighbour Kaplan-Meier***⁺*** |
| Omurlu et al. (2009) | - Cox Model | - RSF log-rank - RSF conservation of events - RSF log-rank score - RSF approximate log-rank |  |
| Steingrimsson and Morrison (2020) | - Cox Model -true main effects - Penalized L1 Cox model | - Doubly robust CUDL***⁺*** - Buckley James CUDL***⁺*** - RSF log-rank - Doubly Robust RSF - Buckley James RSF - DeepSurv |  |
| Wang and Li (2019) | - Penalized L1 Cox model - Penalized L2 Cox model | - ELMCoxBAR ***⁺*** - RSF log rank - RSF Maximally selected Rank - RSF C-index | - CoxBoost |
| Xiang et al. (2000) | - Cox Model – true interactions included | - Faraggi Simon neural network - Liestol-Andersen-Andersen neural network - Buckley-James neural network |  |
| ** Two SuperLearner algorithms were developed and compared by Golmakani and Polly (2020)*  *⁺ denotes a method that was developed by the authors* | | | |
